# Multifunctional near-infrared light-triggered biodegradable micelles for chemo- and photo-thermal combination therapy

**DOI:** 10.18632/oncotarget.10320

**Published:** 2016-06-29

**Authors:** Jie Cao, Dan Chen, Shanshan Huang, Dawei Deng, Liping Tang, Yueqing Gu

**Affiliations:** ^1^ Department of Biomedical Engineering, State Key Laboratory of Natural Medicines, School of Engineering, China Pharmaceutical University, Nanjing, China; ^2^ Department of Bioengineering, University of Texas at Arlington, Arlington, Texas, USA

**Keywords:** near-infrared light-triggered nanomicelles, photo-thermal therapy, chemotherapy, tumor targeting, paclitaxel

## Abstract

A combination of chemo- and photo-thermal therapy (PTT) has provided a promising efficient approach for cancer therapy. To achieve the superior synergistic chemotherapeutic effect with PTT, the development of a simple theranostic nanoplatform that can provide both cancer imaging and a spatial-temporal synchronism of both therapeutic approaches are highly desired. Our previous study has demonstrated that near-infrared (NIR) light-triggered biodegradable chitosan-based amphiphilic block copolymer micelles (SNSC) containing light-sensitive 2-nitrobenzyl alcohol and NIR dye cypate on the hydrophobic block could be used for fast light-triggered drug release. In this study, we conjugated the SNSC micelles with tumor targeting ligand c(RGDyK) and also encapsulated antitumor drug Paclitaxel (PTX). The results show that c(RGDyK)-modified micelles could enhance the targeting and residence time in tumor site, as well as be capable performing high temperature response for PTT on cancer cells and two-photon photolysis for fast release of anticancer drugs under NIR irradiation. *In vitro* release profiles show a significant controlled release effort that the release concentration of PTX from micelles was significantly increased with the exposure of NIR light. *In vitro* and *in vivo* antitumor studies demonstrate that, compared with chemo or PTT treatment alone, the combined treatment with the local exposure of NIR light exhibited significantly enhanced anti-tumor efficiency. These findings indicate that this system exhibited great potential in tumor-targeting imaging and synchronous chemo- and photo-thermal therapy.

## INTRODUCTION

Multifunctional nanomaterials, where therapeutic and imaging features are integrated within a single nanoplatform, has offered a promising opportunity for concurrent diagnosis and treatment of cancer tumors [1–3]. Up to now, cancer therapy relying on an individual therapeutic modality is generally undesirable; usually causing incomplete tumor destruction thus allows tumor regrowth in the long term. Combination therapy based on two or more different therapeutic mechanisms provides a highly efficient approach to treat neoplastic tissues [4]. Recently, nanoparticles with photo-thermal therapeutic capabilities have attracted tremendous attention in the treatment of tumor cells [5–7]. PTT destroys cancer cells with minimal invasiveness by generating the localized heat within a tumor site upon the absorption of specific light wavelength [8]. Those near-infrared (NIR) resonant nanomaterials are chosen due to the optimal transmission to tissue in the NIR region. However, there are still challenges for clinical applications. Some studies have shown that PTT alone will not destroy all malignant cells and may allow residual cells to survive after photo-thermal injury. To overcome this limitation, combined PTT with chemotherapy in a single platform is expected to generate a synergistic effect and decrease cancer recurrence rates [9–13]. The addition of imaging abilities to such a combinatorial modality also allows the identification of microscopic cancer tumors for a more precise and highly efficient treatment [14]. Currently, the widely studied nanocarriers for chemo-photo-thermal therapy are mostly based on mesoporous silica-coated gold or carbon materials [15, 16]. However, the drug release profiles cannot be controlled precisely and only a limited amount of drug can be released in these systems over a long period even at an elevated temperature. It may prevent the superior synergistic chemotherapeutic effect with PTT. Consequently, the development of a simple theranostic nanoplatform that can provide both cancer imaging and a spatial-temporal synchronism of PTT and chemotherapy are highly desired.

Increasing interest has been showed on developing nanomedicine for cancer therapy by designing nanomicelles capable of carrying therapeutic agents to the target tissues/organs while possessing triggered drug release property. Stimuli-responsive nanocarriers (pH-, thermo-, ultrasound-, enzyme- and light-sensitive) which could control micelle dissociation and triggered drug release in response to the pathological environment-specific stimuli and/or externally applied signals provide advantages of regulating the therapy process both spatially and temporally [17–22]. Among them, light-responsive micelles have become a favorable choice that can be disrupted by illumination. It is conceivable that if the release of encapsulated agents is triggered by light, the time and the location of release can determined by applied irradiation [23–26]. Because most of the light-triggered nanocarriers are activated by UV and visible light, while the photo-thermal agents are activated by a NIR laser, the combination of light-triggered chemotherapy and PTT needs two different light wavelengths. The sequential irradiation based on two lasers prolongs the treatment time and requires precise alignment of the two light beams. In addition, the UV/Vis light may cause skin damage and limit its deep tissue application. Hence, it remains challenging to develop a platform for combined PTT and chemotherapy that can be synchronously controlled by a single NIR laser with controlled drug release. Our previous study has successfully constructed a biocompatible near-infrared light-breakable chitosan-based amphiphilic block copolymer micelles (SNSC) containing light-sensitive 2-nitrobenzyl alcohol on the hydrophobic block for drug delivery [27]. Two-photon photolysis of the micelles could be triggered by NIR light (765 nm). It is demonstrated that, once the release of encapsulated agents is triggered by light, the time and the duration of release could be controlled by applied near-infrared irradiation. To accelerate the dissociation of the micelles under NIR illumination, a NIR dye cypate (Ex/Em: 780/808 nm) was encapsulated into the hydrophobic core of the micelles. When irradiated by NIR light (765 nm), the emission light (808 nm) of cypate could be re-absorbed by the 2-nitrobenzyl groups in the core of micelles, accelerating the dissociation of the hydrophobic core to lead the collapse of the micelles. In this case, a dual NIR emission made the two-photon cleavage reaction faster, resulting in faster dissociation of the nanomicelles and faster concomitant release of co-loaded hydrophobic species.

Despite intensive research efforts on developing light-sensitive nanocarriers for cancer theranostics, the use of such drug-loaded micelles with active-tumor-targeting capability, as well as PTT-chemotherapy combination for tumor treatment has not been fully explored yet. Hence, in this study, to get the highest drug concentration to the tumor tissue as possible, we developed a multifunctional micellar drug delivery platform with the capability of active tumor targeting, NIR light-triggered anticancer drug release for two-photon stimulated combination of PTT and chemotherapy, as well as NIR imaging. The nanoplatform was designed for co-loading cypate as an imaging agent as well as a photo-thermal agent, and paclitaxel (PTX) as a chemotherapeutic drug into the inner core of the SNSC micelles, with cyclic RGD as an active targeting ligand conjugated to the surface of the micelles to increase the selectivity for tumor cells and enhance intracellular drug delivery, while reducing systemic toxicity and adverse side effects compared to untargeted micelles and systemic chemotherapy.

## RESULTS AND DISCUSSION

### Synthesis and characterization of c(RGDyK)-SNSC-cypate-PTX

**Figure 1:**
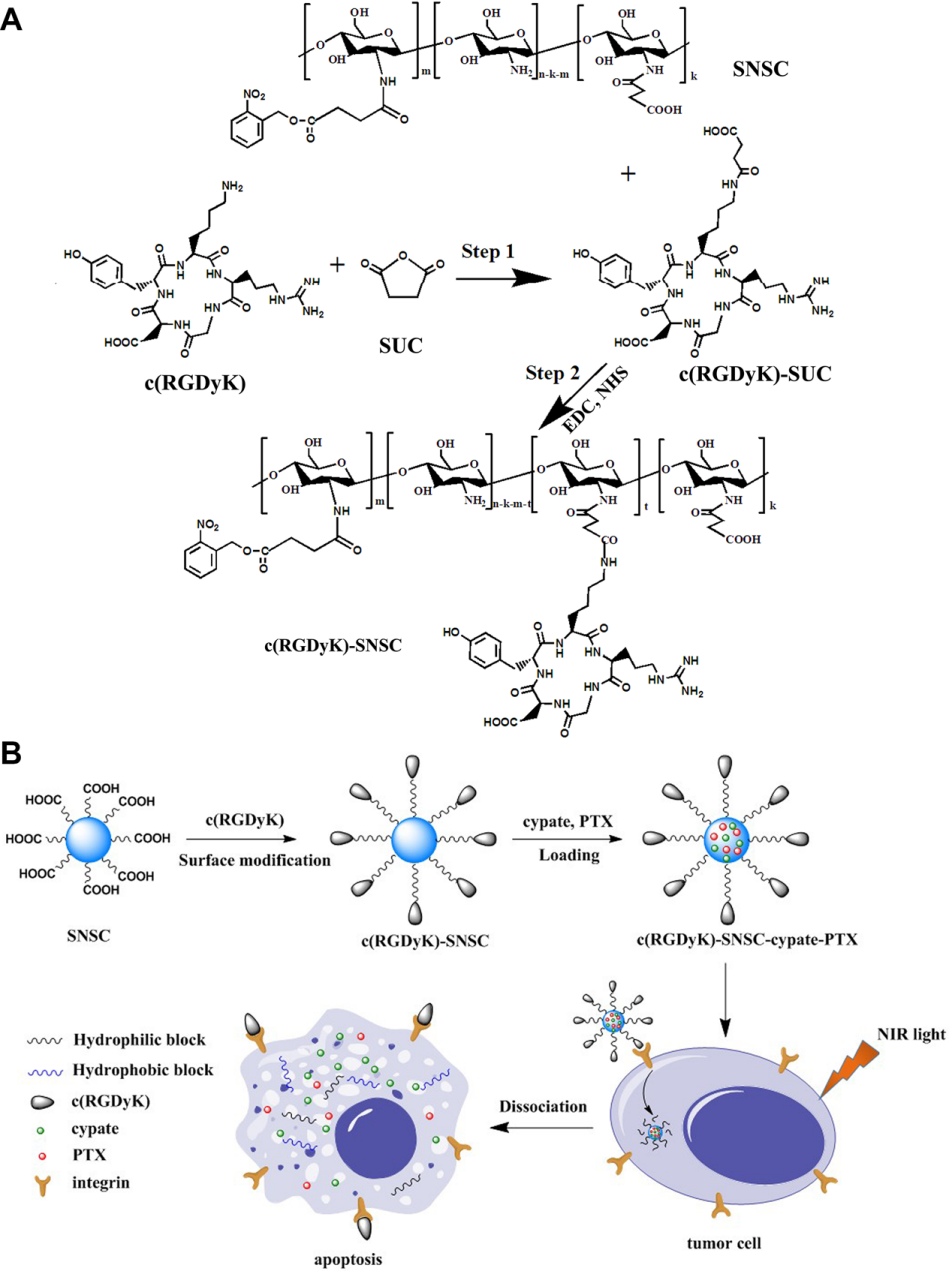
Synthetic scheme and structures of targeted NIR light sensitive nanoplatform c(RGDyK)-SNSC and α_v_β_3_-mediated binding of tumor cells

As described in the method section, the cyclic RGD was reacted with succinyl anhydride to form amide bond. The carboxyl group was activated by EDC and NHS system and then reacted with the amino group of NIR triggered chitosan micelles to synthesize tumor-targeted c(RGDyK)-modified SNSC micelles (Figure 1A). The mass spectrometry of the conjugation of cyclic RGD and succinyl anhydride was shown in Figure S1A. The peak of 704.2 proves the successful conjugation of succinyl anhydride (MW_c(RGDyK)-SUC_ = 704.3). Furthermore, by comparing the FTIR spectra of SNSC and c(RGDyK)-SNSC (Figure S1B), the sharpening and strengthening of the 3427.5 cm^−1^ peak is attributed to stretching vibration of secondary amides and free hydroxyl groups of c(RGDyK). The increasing of carboxyl groups of the compound made the peak 1675.9 cm^−1^ strengthen and the intensified in-plane bending vibration peak of 1527.6 cm^−1^ was shown in Figure S1B. In addition, the vibration of the benzene ring skeleton supports the successful conjugation of c(RGDyK) with benzene ring and series of -NH.

**Figure 2:**
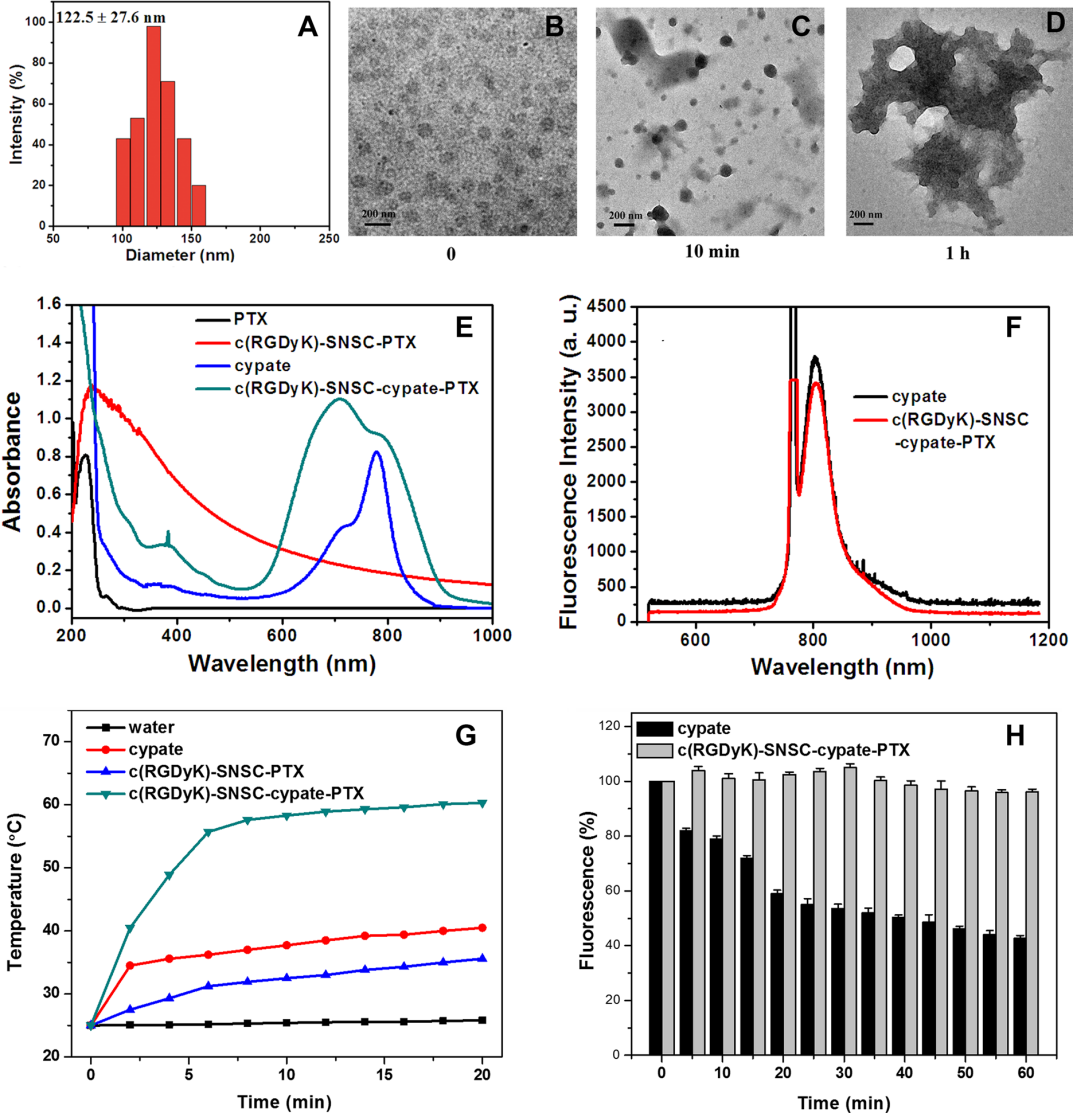
Size distribution and optical properties of c(RGDyK)-SNSC-cypate-PTX (**A**) Size distribution of c(RGDyK)-SNSC-cypate-PTX micelles; (**B**) Transmission electron microscope pictures of c(RGDyK)-SNSC-cypate-PTX; TEM images of c(RGDyK)-SNSC-cypate-PTX after NIR illumination (765 nm, 400 mW/cm^2^) for 10 min (**C**) and 1 h (**D**). (**E**) Absorption spectra of PTX, c(RGDyK)-SNSC-PTX, cypate and c(RGDyK)-SNSC-cypate-PTX; (**F**) Photoluminescence spectra of cypate and c(RGDyK)-SNSC -cypate-PTX; (**G**) Temperature change curves of 500 μL water (black), free cypate in DMSO (red), c(RGDyK)-SNSC-PTX (blue) and c(RGDyK)-SNSC-cypate-PTX (green) exposed to NIR light (765 nm, 400 mW/cm^2^) for 20 min; (**H**) Photostability of cypate and c(RGDyK)-SNSC-cypate-PTX, which was obtained by irradiation of cypate or c(RGDyK)-SNSC-cypate-PTX under NIR light (765 nm, 400 mW/cm^2^).

As described, cypate (Ex/Em: 780/808 nm) and PTX were encapsulated into the hydrophobic core of the micelles to form nanostructure c(RGDyK)-SNSC- cypate-PTX. When the molar ratio of PTX and drug-loading system was 1:4, the micelles exerted the best drug loading property with highest drug loading content (DLC) and encapsulation efficiency (EE) (Table 1). According to the size measurement, the hydrodynamic diameter of c(RGDyK)-SNSC-cypate-PTX was 122.5 nm (122.5 ± 27.6 nm, mean ± standard deviation) (Figure 2A). Thus, the nanocarriers have a hydrodynamic size within the desired range of 10-200 nm to prevent elimination by the kidneys (> 10 nm) and enhance tumor-targeted delivery via the EPR effect (< 200 nm). The NIR-triggered destruction of c(RGDyK)-SNSC-cypate-PTX was observed by TEM (Figure 2B–2D). As shown, c(RGDyK)-SNSC-cypate-PTX micelles were dispersed nearly spherical with an average diameter of around 100 nm (Figure 2B). After illumination by a NIR laser (765 nm, 400 mW) for 10 min, most of the particles were aggregated into larger condensed nanospheres possibly following hydrophobic interaction, while some parts collapsed under the irradiation (Figure 2C). The micelles were completely destroyed after 1 h irradiation (Figure 2D), making the platform useful for light-controlled drug release.

**Table 1:** The DLC and EE of c(RGDyK)-SNSC-PTX and c(RGDyK)-SNSC- cypate-PTX (*n* = 3)

Sample	DLC%	EE%
c(RGDyK)-SNSC-PTX	14.17 ± 0.43	64.37 ± 2.17
c(RGDyK)-SNSC-cypate-PTX	10.26± 0.58	54.86 ± 2.35

### Photo-physical properties of c(RGDyK)-SNSC-cypate-PTX

The distinct absorption and fluorescence spectra of different components in the nanocarrier further confirmed the conjugation of c(RGDyk) and encapsulation of cypate and PTX onto the light-sensitive nanocarrier. The adsorption spectra (Figure 2E) illustrated characteristic absorption peak of PTX and cypate in c(RGDyK)-SNSC micelles at 227 nm and 700–780 nm, respectively. It should be mentioned that absorption spectrum of c(RGDyK)-SNSC-cypate-PTX had broad peak from 700 to 800 nm, due to the formation of aggregates such as H-aggregates in aqueous conditions when compared to monomeric cypate (blue line). The photoluminescence measurement (Figure 2F) showed overlapping emission profile between cypate and c(RGDyK)-SNSC-cypate-PTX, inferring that encapsulation of PTX had no significant influence on the optical property of cypate. In the development of an effective cypate-based theranostic agent, strong NIR absorption and distinct fluorescence emission are required. However, the extensive aggregation of cypate in aqueous media is a major factor in the quenching of their excited state. The obtained data revealed that encapsulation of cypate within the hydrophobic interior of SNSC micelles decrease cypate aggregation and preserves fluorescence intensity after dispersion in water solution.

Our previous work have demonstrated that under 765 nm light irradiation, the emission light (808 nm) of cypate could be re-absorbed by the 2-nitrobenzyl group in the core of SNSC micelles, accelerating the dissociation of the hydrophobic core to lead the collapse of the micelles. In this study, to further investigate the effect of cypate on fast photocleavage of nanomicelles, we measured the temperature changes of c(RGDyK)-SNSC-cypate-PTX under NIR light irradiation (Figure 2G). The temperature profile of c(RGDyK)-SNSC-cypate-PTX had a quick rising phase during the first 6 min followed by a sustained plateau at 60°C. Under the same experimental conditions, the water control showed a temperature increase of only 1.0°C. The c(RGDyK)-SNSC-PTX solution showed a slow increase rate, mainly due to the photoreaction of 2-nitrobenzyl is a exothermal reaction under NIR light irradiation. These results clearly clarify that cypate can efficiently convert absorbed light energies into heat and raise the temperature of the surrounding medium. Free cypate dissolved in DMSO was able to increase the temperature of the solution to only 40°C, which indicate that encapsulation of cypate into the micelles interior and J-aggregate formation significantly improve its photothermal properties in comparison with free cypate [28]. It is noted that raising and maintaining an elevated temperature at tumor site is one of the key factors for an efficient photothermal therapy as protein denaturation, disruption of the cellular membrane and ablation of tumor tissues occur at temperature > 40–43°C. Based on the data we obtained, the c(RGDyK)-SNSC-cypate-PTX could increase the local temperature significantly enough to cause irreversible photothermal damage to the tumor cells. Hence, intense fluorescence and photothermal properties of cypate provide a significant potential for application in both fluorescence bioimaging and photothermal therapy in a fast NIR light-triggered drug release nanocarrier in deep tissues.

The photostability of c(RGDyK)-SNSC-cypate- PTX was further evaluated and compared to cypate. Both c(RGDyK)-SNSC-cypate- PTX and cypate solution were continuously irradiated by a semiconductor laser at 765 nm (light intensity = 400 mW) and their photoluminescence emission intensities were monitored over a period of 60 min by a S2000 eight-gap optical fiber spectrometer. As shown in Figure 2H, the emission intensity of cypate dropped rapidly in the first 20 min, and then decreased gradually to 41.2% of its initial value within 60 min, which is consistent with previous observations [29]. In contrast, the photostability of cypate was significantly enhanced after being entrapped by c(RGDyK)-SNSC under the same experimental conditions. Over 60 min, the photoluminescence (PL) emission of c(RGDyK)-SNSC-cypate-PTX was relatively stable. The enhancement of the photostability indicates a highly stable optically as well as thermally, making the real-time monitoring of the dynamic process of NIR light-sensitive micelles in mouse model for a relatively longer time.

### *In vitro* study

### Cell uptake of c(RGDyK)-SNSC-cypate-PTX

**Figure 3:**
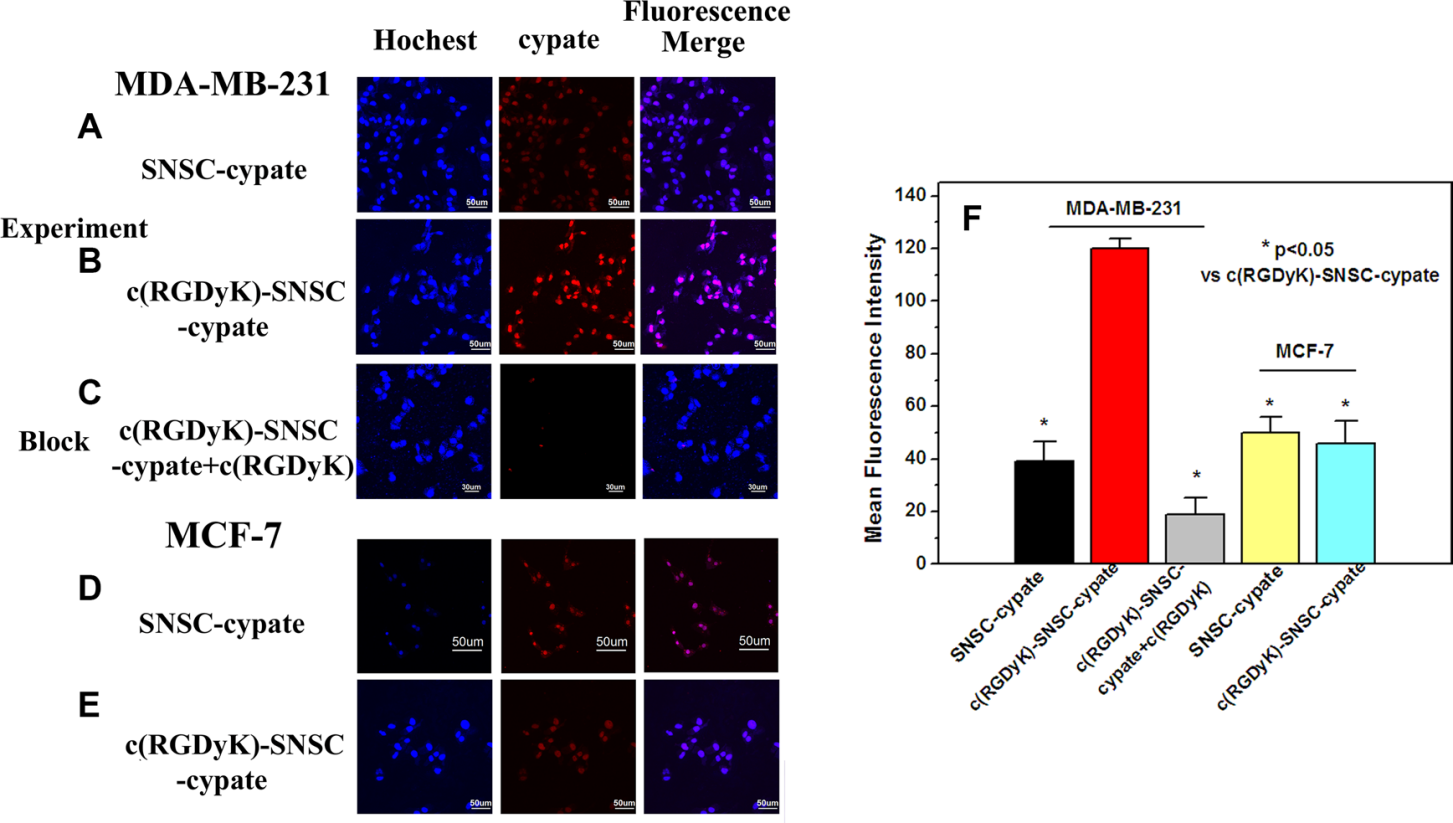
The laser confocal fluorescence microscopy images of α_v_β_3_-positive MDA-MB-231 and α_v_β_3_-negative MCF-7 cells incubated with SNSC-cypate (**A**, **D**), c(RGDyK)-SNSC-cypate (**B**, **E**) at 37°C for 8 h; (**C**) Blocking experiments of c(RGDyK)-SNSC-cypate in MDA-MB-231 cells in the presence of c(RGDyK) (50 mmol/L); Scale bars correspond to 50 μm. (**F**) Mean fluorescence intensity of MDA-MB-231 and MCF-7 cells, incubated with SNSC-cypate or c(RGDyK)-SNSC-cypate in the absence or presence of c(RGDyK). Data are given as mean ± SD (*n* = 5). *P < 0.05.

To evaluate the tumor targeting capability of micelles, integrin α_v_β_3_-positive (MDA-MB-231) and negative (MCF-7) cell lines were used [29]. After incubated with SNSC-cypate or c(RGDyK)-SNSC-cypate for 8 hours, the images of MDA-MB-231 and MCF-7 cells were taken by laser confocal fluorescence microscope (Figure 3). The blue image showed cell nucleus counter stained with Hochest and the red image showed the intracellular micelles with encapsulated cypate. As shown, we found the MDA-MB-231 cells associated with c(RGDyK)-SNSC -cypate showed brighter fluorescence than that with SNSC-cypate (Figure 3A and 3B). In addition, the merged fluorescence images indicate that both SNSC-cypate and c(RGDyK)-SNSC-cypate are mainly located within the cytoplasm region rather than in nuclei, which can be clearly observed by staining the cell nuclei with Hochest. The enhanced cellular uptake of c(RGDyK)-SNSC-cypate is a clear evidence of its tumor-targeting ability. In contrast, with low expression of integrin α_v_β_3,_ MCF-7 cells have a very weak affinity to both SNSC-cypate and c(RGDyK)-SNSC-cypate (Figure 3D and 3E). These results confirmed that c(RGDyK)-SNSC-cypate target cancer cells mainly by triggering the receptor mediated endocytosis of the micelles.

The specificity of c(RGDyK)-SNSC-cypate uptake via integrin α_v_β_3_ was conducted and displayed in Figure 3C. As shown, the uptake of c(RGDyK)-SNSC- cypate was significantly inhibited by c(RGDyK) (50 mmol/L) in the MDA-MB-231 cells. The corresponding mean fluorescence intensity of the MDA-MB-231 cells incubated with c(RGDyK)-SNSC-cypate in the absence and presence of c(RGDyK), as shown in Figure 3F, further indicated that the uptake was mostly mediated by integrin α_v_β_3_. Therefore, the enhanced tumor uptake of c(RGDyK)-SNSC-cypate has potential to increase delivered anticancer drug amount to the tumor sites.

### *In vitro* antitumor efficacy of c(RGDyK)-SNSC-cypate-PTX

**Figure 4:**
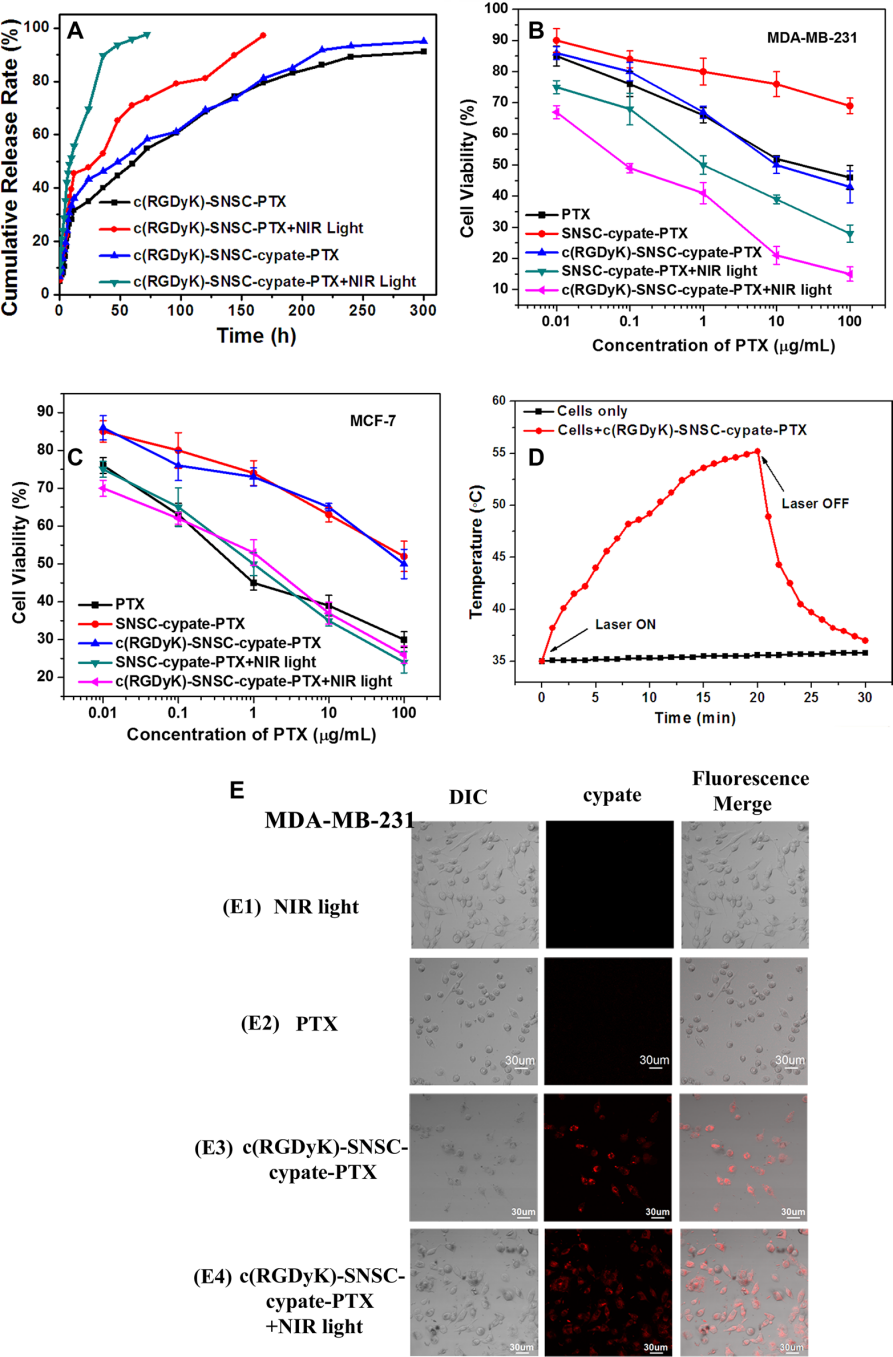
(**A**) Cumulative release rate of paclitaxel (PTX) from c(RGDyK)-SNSC-PTX and c(RGDyK)-SNSC-cypate-PTX micelles with and without NIR illumination (Ex: 765 nm, 800 mW/cm^2^). The cell viability of (**B**) α_v_β_3_-positive MDA-MB-231 cells and (**C**) α_v_β_3_-negative MCF-7 cells were determined following the incubation with free PTX (as control), and SNSC micelles loaded with various PTX concentration (0.01, 0.1, 1, 10, and 100 μg/ml) with or without NIR irradiation (Ex: 765 nm, 800 mW/cm^2^, 20 min). (**D**) Dynamic temperature profile of MDA-MB-231 cells transfected with c(RGDyK)-SNSC-cypate-PTX and exposed to NIR laser light (Ex: 765 nm, 800 mW/cm^2^). Non-treated cells exposed to NIR light were as control. The arrows indicate when the laser turned on and off, respectively. (**E**) Cell uptake and morphology after incubating PTX or c(RGDyK)-SNSC-cypate-PTX with or without NIR irradiation (Ex: 765 nm). *n* = 5 for all groups.

Before investigating the *in vitro* antitumor efficacy of c(RGDyK)-SNSC- cypate-PTX, the cumulative release profile of PTX from c(RGDyK)-SNSC-PTX and c(RGDyK)-SNSC-cypate-PTX micelles in the presence or absence of NIR illumination was studied (Figure 4A). As shown, due to the adsorption of PTX to the surface of the micelles during the synthetic process, the drug release profiles of all the test samples displayed similar two-phase profiles, with initial burst release followed by a slower release regardless of NIR light exposure. However, the release of PTX from c(RGDyK)-SNSC-cypate or c(RGDyK)-SNSC was substantially increased with NIR illumination. Specifically, 69.6% of encapsulated PTX was released from c(RGDyK)-SNSC-cypate while 47.59% was released from c(RGDyK)-SNSC under NIR light irradiation (765 nm, 800 mW) for the first 20 min. In contrast, only 43.29% of encapsulated PTX was released from c(RGDyK)-SNSC-cypate and 34.2% from c(RGDyK)-SNSC without NIR exposure at the first 20 min, respectively. As expected, the PTX release from c(RGDyK)-SNSC-cypate under NIR irradiation is much faster than that from c(RGDyK)-SNSC with or without NIR irradiation. More than 95% of PTX released from c(RGDyK)-SNSC-cypate with NIR irradiation at 30 h post-dialysis. It should be mentioned that the release of PTX from both c(RGDyK)-SNSC-cypate and c(RGDyK)-SNSC is much slower without NIR irradiation. This may due to the strong binding force of PTX to the micelle because of its high hydrophobicity. The release profiles support that the release of PTX from c(RGDyK)-SNSC could be accelerated by co-loading with cypate under NIR illumination, which could shorten treatment period and reduce side effects on normal tissues as well as increase its efficacy in tumors.

To evaluate the combinatorial photothermal therapy and chemotherapy efficacy of c(RGDyK)-SNSC-cypate-PTX, cell viability assays were carried out in cancer cells (MDA-MB-231 and MCF-7). As anticipated, the higher the drug loading concentrations were, the stronger the growth inhibition effect exerted (Figure 4B and 4C). We revealed the high efficacy of combinatorial PTT and chemotherapy of c(RGDyK)-SNSC-cypate-PTX under NIR irradiation in integrin-positive MDA-MB-231 cells, with more than 85% cells deaths when the concertration of PTX reached 100 μg/mL (Figure 4B), in comparison with PTX (45.87 ± 1.53% cell viability), c(RGDyK)-SNSC-cypate-PTX without irradiation (30.02 ± 1.76% cell viability), and SNSC-cypate-PTX with (28.13 ± 1.8 % cell viability) or without NIR irradiation (69.4 ± 0.82% cell viability) at the same PTX concentration. The NIR irradiation could make the dissociation of SNSC nanocarrier faster and accelerate concomitant release of PTX. The modification by cyclic peptides c(RGDyK) of drug carrier increased the targeting capability for integrin-positive tumor cells and enhanced cell uptake to improve antitumor efficacy. In contrast, without c(RGDyK) modification and NIR irradiation, SNSC-cypate-PTX showed the worst antitumor efficacy, mainly due to slow drug release and low cell uptake without integrin targeting. Consequently, our results support the following conclusions: (1) The conjugation of c(RGDyK) increased the cell targeting and growth inhibition of SNSC nanocarriers; (2) The inhibitory effect increased with NIR illumination.

Similarly, the samples inhibitory effect on MCF-7 cells was enhanced by NIR irradiation (Figure 4C). Compared with the samples without NIR illumination, SNSC nanocarrier with NIR illumination showed higher efficacy supporting that these micelles released more drug in accord with the results of *in vitro* drug release. The results also inferred that there was no significant difference of inhibitory effects on integrin-negative cells between SNSC-cypate-PTX and c(RGDyK)-SNSC-cypate- PTX regardless of NIR irradiation, further confirmed the cell uptake of c(RGDyK)-SNSC-cypate-PTX was mediated by integrin receptor.

To further confirm the efficacy of c(RGDyK) targeted NIR illuminated PTX-loaded SNSC micelles in tumor cells, the cell uptake and cell morphology were displayed after incubated in MDA-MB-231 cells for 6 h (Figure 4E), with cells only treated with NIR light for 6 h as control. As shown, there's no obvious change of cell morphology in the control group (Figure 4E1). However, the morphologic characteristics of apoptosis cells, such as cell shrinkage, and fragmentation were observed after the cells incubated with PTX (Figure 4E2), c(RGDyK)-SNSC-cypate-PTX with or without NIR irradiation. Especially, the cells incubated with c(RGDyK)-SNSC-cypate-PTX (Figure 4E4) after NIR light showed more apoptosis, indicating the fast cleavage of SNSC micelles after NIR illumination and the combinatorial efficacy of PTT and chemotherapy of c(RGDyK)-SNSC-cypate-PTX. These results were consistent with MTT assay, further confirmed the effectiveness of our c(RGDyK) targeted NIR light-triggered micelles for integrin positive tumor cells.

The temperature measurement was also performed to confirm the PTT mechanisms was activated under exposure of c(RGDyK)-SNSC-cypate-PTX-treated cancer cells to 765 nm laser light of 800 mW power density. After exposure, the MDA-MB-231 cells incubated with c(RGDyK)-SNSC-cypate-PTX exhibit rapid heating with a starting temperature of 35°C. The maximum temperature reached as high as 55°C (Figure 4D, red curve), while the temperature change for non-transfected cells was less than 1°C (Figure 4D, black curve). Besides, the achieved cellular temperature of cells incubated with c(RGDyK)-SNSC-cypate-PTX continuously increased during the required periods of time under NIR light exposure. However, switching off the laser resulted in the rapid cooling of cells to 37°C in 10 min. The above data clearly indicate that c(RGDyK)-SNSC-cypate-PTX perform a high efficacy to cancer cells in the presence of NIR light, which have potential to minimize side effects and maximize delivery of the active component to the targeted tumors *via* both EPR effect and integrin-receptor.

### *In vivo* study

### *In vivo* targeting ability

**Figure 5:**
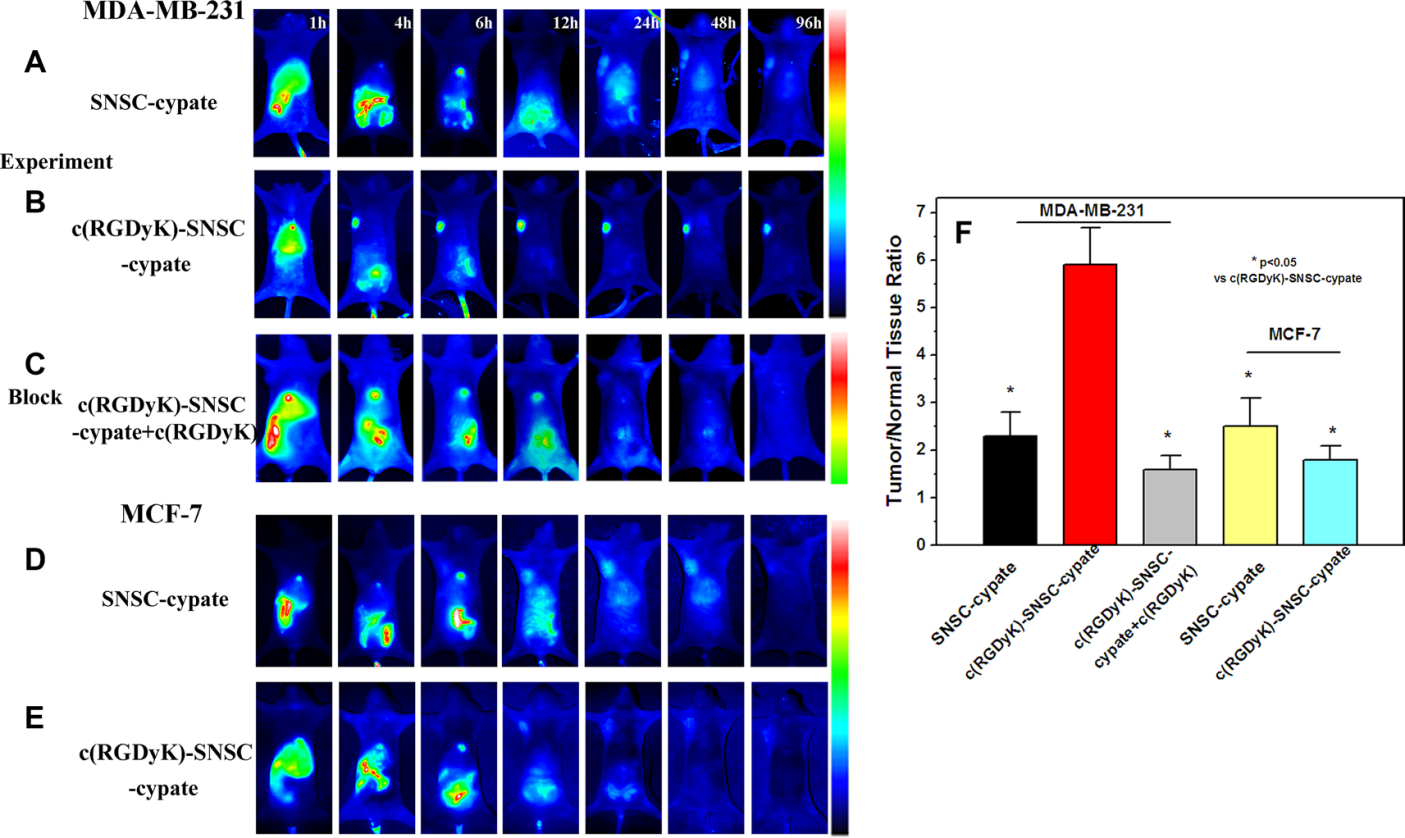
*In vivo* targeting behavior of c(RGDyK)-SNSC micelles in tumor bearing animals for 1 to 96 hours Fluorescence images of α_v_β_3_-positive MDA-MB-231 tumor-bearing mice after systemic injection of (**A**) SNSC-cypate and (**B**) c(RGDyK)-SNSC-cypate micelles; (**C**) c(RGDyK)-SNSC-cypate micelles with blocking dose of c(RGDyK). In comparison, Fluorescence images of αvβ3-negative MCF-7 tumor-bearing mice after injection of (**D**) SNSC-cypate and (**E**) c(RGDyK)-SNSC-cypate micelles. (**F**) Tumor/normal tissue ratio (T/N ratio = [tumor signal – background signal]/[normal signal (muscle) – background signal] × 100%) calculated from the ROIs at 24 h post-injection of SNSC-cypate and c(RGDyK)-SNSC-cypate micelles with or without blocking dose of c(RGDyK). Data are given as mean ± SD (*n* = 5). *P < 0.05.

After *in vitro* results, we constructed *in vivo* studies to evaluate the NIR imaging targeting ability and therapeutic efficacy of the newly developed theranostic nanoplatform. The *in vivo* targeting ability c(RGDyK)-SNSC-cypate was assessed in mice implanted with α_v_β_3_-positive MDA-MB-231. As shown in Figure 5A, the tumor region of nude mice injected with non-c(RGDyK)-modified SNSC (SNSC-cypate) appeared with some fluorescence signal. The signal reached its maximum 48 h after injection, and then cleared from the tumor site at around 96 h. The weak fluorescent signal at the tumor site is possibly attibuted to EPR effect, specifically, nanoparticles detained passively in tumor tissue via hyper-permeability or leakage of tumor blood vessel. When MDA-MB-231 bearing nude mice were treated with c(RGDyK)-SNSC-cypate, the micelles were accumulated in the tumor region 4 h after injection, and the fluorescence signal continuously increased with maximal accumulation at around 24 h (Figure 5B). The fluorescent signal remained strong in the tumor tissue for more than 96 h and can be explained by receptor clustering. Also, a maximum tumor to normal tissue NIR fluorescence ratio of 5.76 ± 0.85 was attained at 24 h post injection of c(RGDyK)-SNSC-cypate compared with fluorescence ratio of 2.31± 0.56 for SNSC-cypate treated mice (Figure 5F). Furthermore, *in vivo* blocking experiment was conducted and displayed in Figure 5C. As shown, the tumor uptake of c(RGDyK)-SNSC-cypate was significantly inhibited by c(RGDyK) in the MDA-MB-231 tumor bearing mice. The corresponding tumor/normal tissue ratio was 1.57 ± 0.29. In contrast, the results in α_v_β_3_-negative MCF-7 bearing mice treated with SNSC-cypate or c(RGDyK)-SNSC-cypate (Figure 5D and 5E) showed substantially weaker florescent signal at the tumor sites, with the tumor to normal tissue fluorescence ratio of (2.5 ± 0.52, 1.83 ± 0.3) (Figure 5F), supporting that the increasing distribution of c(RGDyK)-SNSC-cypate in tumor tissue was mediated by the active targeting capability of integrin receptor.

### *In vivo* combination antitumor efficacy

**Figure 6:**
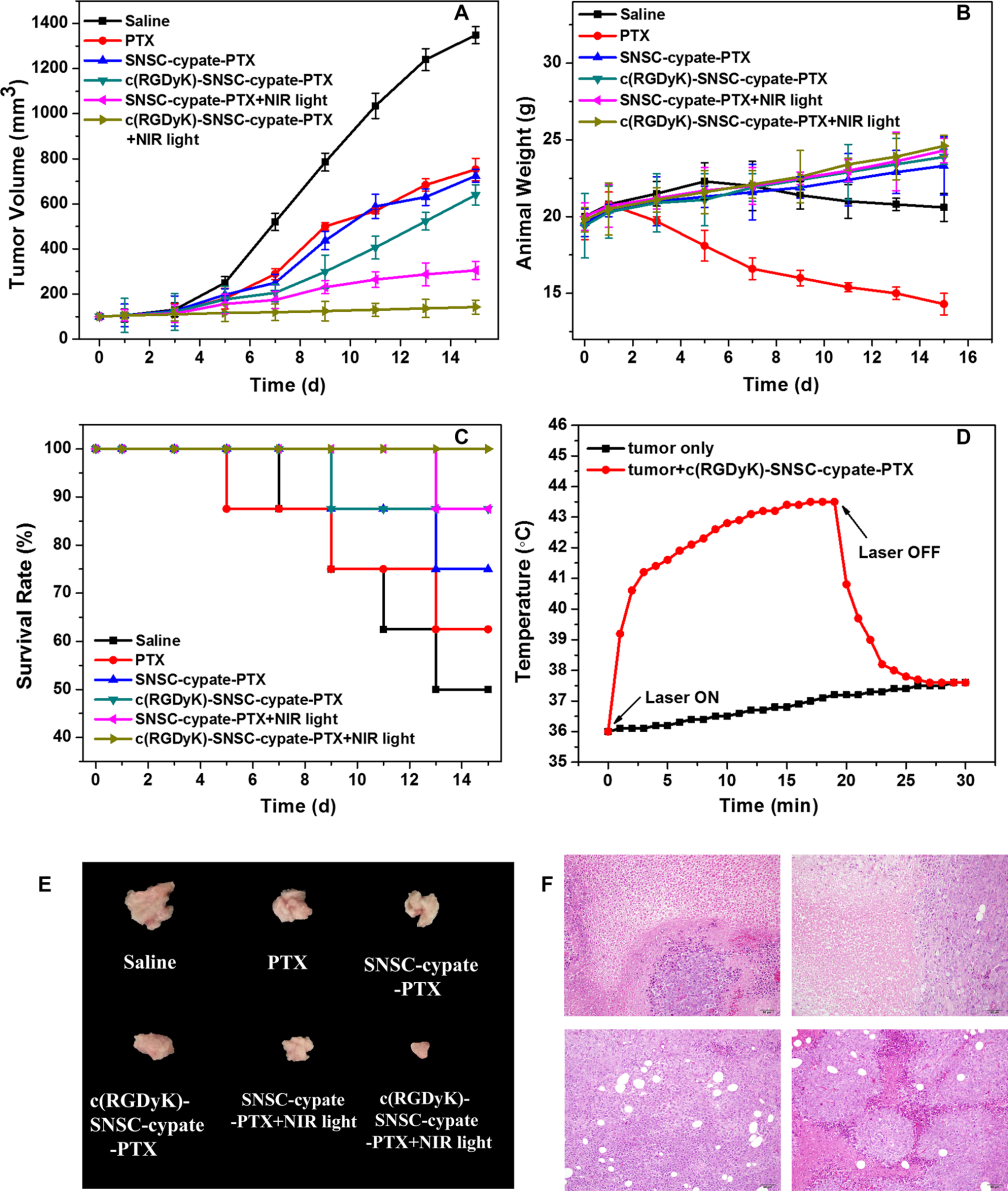
Combinatorial PTT and chemotherapy efficacy of various samples in eradicating MDA-MB-231 tumors implanted in nude mice was determined Test samples include saline (as negative control), PTX (as control), SNSC-cypate-PTX (with or without NIR light irradiation), c(RGDyK)-SNSC-cypate-PTX (with or without NIR light irradiation). Various treated micelles and controls were administered via intravenous injection and the therapeutic effect of various samples was determined by measuring (**A**) tumor volumes, (**B**) animal weight, (**C**) 14-day survival rates of MDA-MB-231 tumors-bearing mice, and (**E**) tumor size of the mice with different samples sacrificed after 14-day post-injection. (**D**) Temperature changes inside the tumor treated with c(RGDyK)-SNSC-cypate-PTX and exposed to NIR laser light (Ex: 765 nm, 800 mW/cm^2^). Untreated tumor exposed to NIR light were as control. The arrows indicate when the laser turned on and off, respectively. (**F**) H&E stained tissue samples excised from animals treated with saline (F1), PTX (F2), c(RGDyK)-SNSC-cypate-PTX (without (F3) or with NIR irradiation (F4)) micelles. Image magnification is 200× and *n* = 8 in all groups.

To evaluate the anticancer efficacy of *in vivo* combinatorial PTT and chemotherapy, MDA-MB-231 tumors treated with c(RGDyK)-SNSC-cypate-PTX were exposed for 20 min to a 765 nm laser at a power density of 800 mW. SNSC-cypate-PTX and PTX MDA-MB-231 tumors were as control. The anti-tumor effects of various treatments were then quantified based on tumor volume, animal weight change, and survival rate (Figure 6A–6C). Under irradiation, the intratumoral temperature changes were also recorded. As shown, the temperature increased rapidly from 36°C to 40.5°C within 2 min, reaching 42.7°C plateaus after 10 min (Figure 6D, red curve). In comparison, the temperature of the tumor without c(RGDyK)-SNSC-cypate- PTX treatment had almost no changes under the same 765 nm irradiation conditions (Figure 6D, black curve). Thus, compared with the control group (saline injection), drug-treated groups, including PTX, SNSC-cypate-PTX and c(RGDyK)-SNSC-cypate-PTX with or without NIR illumination significantly reduced MDA-MB-231 tumor volume, among which c(RGDyK)-SNSC-cypate-PTX under NIR illumination exerted the best inhibitory efficacy (Figure 6A), in consistent with the results of tumor size of the mice treated with different samples after 15-day post injection (Figure 6E). There are several potential causes for such effects, namely, 1) c(RGDyK)-modified micelles were positively targeted tumor tissue by integrin receptor, thus increasing the tumor inhibitory chemo-effect of micelles; 2) Under NIR illumination, the cypate entrapped micelles exerted better inhibitory effects on tumor growth. Upon irradiation, cypate in micelles has increased the local temperature significantly enough to cause irreversible photothermal damage to the tumor tissue.

Body weight and survive rate usually reflect the health condition of the treated mice. The treatments have different effects on the changes of animal weight during the therapeutic process (Figure 6B). Animal weight of control saline group reduced after rising first. Weight of free PTX groups reduced by 21.43% after two-week treatment compared with the initial weight, indicating the potential systemic toxicity of PTX. Animal weight of other groups either with or without NIR illumination after two-week treatment increased slowly, supporting the potential therapeutic effect of various micelles. It is likely that micelles can enhance PTX distribution in tumor tissues and reduce distribution in other normal organs, thus reducing side effects of chemotherapy. 15-day survival rates of mice in control and PTX-treated groups reduce to 50 % and 63%, respectively (Figure 6C). None of the mice died in 765 nm irradiated c(RGDyK)-SNSC-cypate-PTX treated group. Survival rates of mice in the SNSC-cypate-PTX with or without NIR irradiation and non-NIR irradiation c(RGDyK)-SNSC-cypate-PTX treated group reach to 75%, 88% and 88%. These results further demonstrated the low cytotoxicity of PTX after encapsulated into micelles and indicated that after irradiation, combinatorial PTT and chemotherapy based on c(RGDyK)-SNSC-cypate-PTX can effectively improve the survival quality of mice and prolong their lifetime.

To further confirm the therapy efficacy of micelles on the subject animals, the tumors were excised one week after micelles treatment for pathological evaluation (Figure 6F). Histological analysis reveals that no damage was found in tumors of control mice (Figure 6F1). However, the treatment of free PTX (Figure 6F2) and administered with c(RGDyK)-SNSC-cypate-PTX without or with irradiation (Figure 6F3, 6F4) showed serious injury in the form of cellular edema and nuclear swelling, with c(RGDyK)-SNSC-cypate-PTX under illumination showed markedly increased apoptotic and necrotic tumor cells, further indicating that the efficacy of the micelles is due to the release of PTX after the dissociation of the micelles under NIR illumination. Besides, cyclic RGD could enhance the accumulation of the micelles, thus increase the anticancer efficacy of the micelles.

## MATERIALS AND METHODS

### Materials

Chitosan was purchased from Aoxing Biotechnology Co. Ltd. (Zhejiang, China), with deacetylation degrees of 90% and an average molecular weight (MW) of 50 kDa. N,N'-dicyclohexylcarbodiimide (DCC), N-hydroxysuccinimide (NHS), 2-nitrobenzyl alcohol, pyridine were all purchased from Sigma-Aldrich (St. Louis, USA). Indocyanine Green (ICG) derivative cypate (MW 689) was prepared in our laboratory. Cyclic RGD (c(RGDyK)) was purchased from Shanghai Jier Biochemistry Co. Methyl thiazolyltetrazolium (MTT), RPMI 1640 medium and fetal bovine serum (FBS), penicillin, streptomycin, trypsin and ethylenediamine tetra-acetic acid (EDTA) were purchased from Invitrogen-Life Technologies (Carlsbad, CA, USA). All other analytical reagent grade chemical reagents used in the study were commercially acquired from Shanghai Chemical Reagent Company (Shanghai, China).

Human breast cancer cell lines (MCF-7), human glioma cell lines (U87MG) and human breast cancer cell lines (MDA-MB-231) were all purchased from American Type Culture Collection (ATCC, Manassas, VA, USA). Kunming normal mice (male and female) and Athymic nude mice (nu/nu CD-1 male and female) were purchased from SLAC Laboratory Animal Co. Ltd. (Shanghai, China).

### Preparation of c(RGDyK)-modified NIR light-triggered chitosan micelles (c(RGDyK)-SNSC)

The c(RGDyK)-modified photoactive amphiphilic diblock copolymer was synthesized as depicted in Figure 1. First, the cyclic peptides c(RGDyK) reacted with succinic anhydride (Step 1). Then the activated product was synthesized with SNSC (Step 2). Specifically, c(RGDyK) (5 mg) and succinic anhydride (3 mg, SUC) were first dissolved in 2 mL DMF and reacted for 18 h at room temperature and the mass spectrum (MS) was scanned to identify the product. Second, the -COOH group of the product was activated with DDC/NHS catalyst systems (molar ratio of c(RGDyK)-SUC:EDC:NHS=1:1.5:1.5) under continuous stirring for 24 h. Then, the activated c(RGDyK)-SUC was dissolved in 1 mL acetic acid and was added dropwise into prepared N-succinyl-chitosan acetic acid solution with stable stirring for another 24 h. Afterwards, the activated light-sensitive group 4-(2-nitrobenzyloxy)-succinyl ester was added to the above acetic acid solution with continuous stirring for another 18 h. After that, the product was purified by dialysis (MWCO 10000) against distilled water for 2–3 d. After dialysis, the solution was self-assembled with sonication at*P* = 300 W for 50 times and then centrifuged. The supernatant c(RGDyK)- N-succinyl-N′-4-(2-nitrobenzyloxy)-succinyl chitosan (c(RGDyK)-SNSC) micelles were kept at room temperature for further research.

**Figure:**
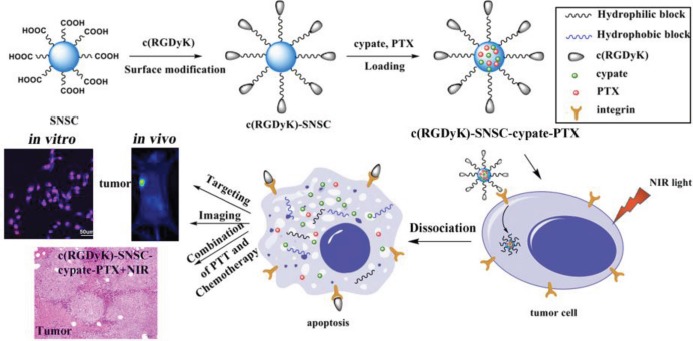
Table of Contents Graphic A multifunctional micellar drug delivery system with the capability of active tumor targeting, imaging and light-triggered release for combination photo-thermal and chemo-therapy was developed.

### Uploading near-infrared dye cypate and antitumor drug paclitaxel (PTX)

Our previous study has shown that the dissociation of the NIR fluorescent dye cypate-encapsulated micelles was accelerated under NIR (765 nm) illumination. The NIR emission triggered the photo-cleavage reaction, resulting in accelerated dissociation of SNSC micelles and concomitant release of co-loaded hydrophobic species. Here, we encapsulated cypate and antitumor drug PTX to the NIR light-triggered c(RGDyK)-SNSC nanocarrier. Specifically, 1 mg cypate dissolved in 100 μL DMSO was added dropwise into 2 mL c(RGDyK)-SNSC or SNSC micelles. Likewise, we added 2 mg PTX of 100 μL DMSO into micelles. The solution was stirred continuously for 10 min and sonicated at 200 W for 30 times. Then the solution was purified by dialysis in a dialysis tube (MWCO 10000) against distilled water and centrifuged (10000 rpm, 10 min) to remove free PTX. The entrapment efficiency and loading content were calculated according to the following Equation: Entrapment efficiency = (mass of drug loaded in micelles/mass of drug fed initially) × 100%; Loading content = (mass of drug loaded in micelles/mass of drug loaded micelles) × 100%.

### Characterization of c(RGDyK)-SNSC-cypate-PTX

The Fourier Transform Infrared (FTIR) spectra of c(RGDyK)-SNSC micelles was recorded by FTIR spectrometer (Nicolet ECO2000, USA). The average diameters and distribution of c(RGDyK)-SNSC, c(RGDyK)-SNSC-cypate and c(RGDyK)-SNSC- cypate-PTX were measured by a Mastersizer 2000 Laser Particle Size Analyzer (LPSA, Malvern, British) with a helium–neon laser (10 mW max, wand wavelength of 633 nm) as the light source at the scattering angle of 90°. The available detecting size interval is 2 nm–3 μm.

### Absorption and fluorescence measurement

The UV-Vis spectra were acquired by 754-PC UV-Vis spectrophotometer (JingHua technological instrument corporation, Shanghai, China). S2000 eight-channel optical fiber spectrographotometer (Ocean Optics corporation, America), and a NL-FC-2.0-763 semiconductor laser (λ = 605 nm or λ = 765 nm, Enlight, China) light were utilized for fluorescence spectra detection. All optical measurements were performed at room temperature.

### Temperature evaluation induced by NIR laser irradiation

A continuous NL-FC-2.0-763 semiconductor laser (λ = 765 nm, Enlight, China) light was employed. To evaluate the lased induced temperature increase, 500 μL of c(RGDyK)-SNSC-cypate-PTX aqueous solution was irradiated under NIR light for 20 min, while the temperature was monitored with a temperature sensor at designated time intervals. In addition, 500 μL of water or cypate were irradiated at the same laser settings for temperature recording.

### Photostability

For the study of photostability, c(RGDyK)-SNSC-cypate-PTX or cypate aqueous solution were continuously irradiated by a semiconductor laser at 765 nm (light intensity = 400 mW) and their photoluminescence emission intensities were monitored over a period of 60 min by a S2000 eight-gap optical fiber spectrometer.

### *In vitro* study

### *In vitro* cell targeting ability of c(RGDyK)-SNSC-cypate

To evaluate the integrin α_υ_β_3_ receptor specificity of c(RGDyK)-SNSC-cypate, integrin α_υ_β_3_ highly expressed tumor cells MDA-MB-231 was used. On the other hand, MCF-7 with low integrin α_υ_β_3_ expression was served as negative control. Briefly, MDA-MB-231 or MCF-7 cells (3 × 10^5^ cells/mL) were seeded at the confocal petri dish and incubated at 37°C for 24 h. 200 μL of SNSC-cypate or c(RGDyK)-SNSC-cypate solution (1 mmol/L) were added into cell culture media. After cultured for 8 h, the adherent cells were washed with PBS thrice and the cells' nucleus were stained with Hochest (12 μg/mL) for 0.5 h and then imaged using laser confocal microscopy.

To confirm the integrin α_υ_β_3_ receptor mediation, *in vitro* receptor blocking experiment with cyclic RGD (c(RGDyK)) was conducted on MDA-MB-231 cells. After cultured the MDA-MB-231 cells at 37°C for 24 hours, c(RGDyK) (50 mmol/L) was preliminarily added to the cells for 30-minute incubation. Subsequently, c(RGDyK)-SNSC-cypate was added to the dishes and cultured for another 8 h. After washing with PBS, the cells were imaged using laser confocal microscopy.

### *In vitro* drug release

The release profiles of paclitaxel (PTX) from c(RGDyK)-SNSC-cypate micelles *in vitro* were tested by a dialysis method. SNSC-cypate-PTX or c(RGDyK)-SNSC- cypate-PTX micelles (2 mL) were dialyzed in dialysis tubes against phosphate buffered saline (PBS) (pH 7.4, 100 mL, containing 0.1% tween 80) at 37.5°C and 30 rpm with or without the exposure of NIR light (765 nm, 800 mW). The released drug in the incubation buffer was collected and the aliquots taken from the dialysate were replaced with fresh PBS (containing 0.1% tween 80) at pre-determined time intervals to keep the volume constant during the assay. Remove water by a freeze-drying method and re-dissolve the drug by methanol for High Performance Liquid Chromatography (HPLC) analysis.

### *In vitro* drug effect of c(RGDyK)-SNSC-cypate-PTX

To assess the *in vitro* drug efficacy of c(RGDyK)-SNSC-PTX, MTT assay was conducted following the standard protocol reported previously [30]. The MDA-MB-231 and MCF-7 were chosen as the target cells. In detail, 200 mL of MDA-MB-231 (or MCF-7) in RPMI 1640 (2.5 × 10^3^ cell) with 10% FCS (serum) was added into each well in a 96-well plate and incubated for 24 h in humidified atmosphere containing 5% CO_2_ at 37.0°C. The culture medium in each well was replaced by 200 mL of RPMI 1640 containing c(RGDyK)-SNSC-cypate-PTX with particular concentrations (0.01, 0.1, 1, 10, 100 μg/mL). The mixture was then exposed to the laser diode (765 nm, 800 mW, 20 min) and the temperature changes were measured by fiber optic temperature probe. After light irradiation, the cells were incubated for another 48 h. Finally, the medium was replaced by 180 mL of fresh RPMI 1640 and 20 mL of MTT solution (5 mg/mL). After incubating for another 4 h, the medium containing MTT was removed from each well and 200 mL of DMSO was added and shaken at room temperature. The optical density (OD) was measured at 570 nm with a Microplate Reader (Biorad, USA). The viable rate could be calculated by the following equation: viable rate = (OD_treated_/OD_control_) × 100%, where OD_treated_ was obtained in the presence of c(RGDyK)-SNSC-cypate-PTX and OD_control_ was obtained in the absence of c(RGDyK)-SNSC-cypate- PTX. Furthermore, the following control groups were employed in the study to evaluate the efficacy of combinatorial of PTT and chemotherapy: untreated cells, cells treated with PTX, SNSC-cypate-PTX, c(RGDyK)-SNSC-cypate-PTX under dark conditions, cells treated with SNSC-PTX, SNSC-cypate-PTX and c(RGDyK)-SNSC-PTX exposed to the laser diode (765 nm, 800 mW, 20 min). In addition, the morphological changes of cells treated with c(RGDyK)-SNSC-cypate-PTX under irradiation were imaged using laser confocal microscopy.

### *In vivo* study

### *In vivo* dynamic distribution and tumor targeting of the nanoconstruct

Thymus defect nude mice (evenly, male and female animals aged 5~6 weeks, weighed 18~22 g, breed in a GLP laboratory) were used in this investigation. For *in vivo* study, tumor cell MDA-MB-231 or MCF-7 suspension (100 μL, about 3 × 10^6^ cells) was injected into the mice subcutaneously after the mice were anesthetized by isoflurane. When the volume of the tumor reaches to 100 ~ 200 mm^3^, they were used for *in vivo* targeting experiments.

The tumor bearing mice were divided into 2 groups (5 mice per group), which was intravenously injected with either SNSC-cypate-PTX solution (0.2 mL, control) or c(RGDyK)-SNSC-cypate-PTX solution (0.2 mL) via the tail vein. To capture the real time dynamics and biodistributions of the c(RGDyK)-SNSC-cypate-PTX in the tumor bearing mice, a custom-constructed NIR imaging system was used. The *vivo* fluorescence images of animal were obtained at designated time intervals (1–96 h).

### *In vivo* combinatorial PTT and chemotherapy effect

The tumor bearing mice were divided into 6 groups (8 mice per group), shown in Table 2. When the volume of tumor reached 100 mm^3^, drug or saline was given every other day. During this period, we recorded the weight and volume of the tumor.

**Table 2:** The tumor bearing mice were divided into 6 groups (A–F) and each group mice were intravenously injected with different samples

	Sample	NIR light irradiation (765 nm, 800 mW, 20 min)
A	Saline	×
B	PTX	×
C	SNSC-cypate-PTX	×
D	c(RGDyK)-SNSC-cypate-PTX	×
E	SNSC-cypate-PTX	√
F	c(RGDyK)-SNSC-cypate-PTX	√

For the mice injected with SNSC-cypate-PTX (Group E) and c(RGDyK)-SNSC-cypate-PTX (F), the NIR laser (765 nm, 800 mW) was applied to the tumors for 20 min post injection.

All of the groups were intravenously injected with samples (5 mg/kg body weight at an equivalent dose of free PTX) via the tail vein, respectively. The E-H group were irradiated by NIR laser (765 nm, 800 mW, 20 min) at 6 h post-injection. Tumor volume (TV) is calculated by the equation: TV = 1/2*a*b^2^, where *a* represents the length of the tumor and *b* represents the width of the tumor. The relative tumor volume (RTV) is found by the equation: RTV = V_t_/V_0_, where V_t_ represents the tumor volume before administration and V_0_ represents the recorded tumor volume. Relative tumor inhibitory rate (T/C) is the assessment of antitumor activity of human xenograft tumor model and is calculated by the equation T/C(%)=TRTV/CRTV×100%, where TRTV is obtained by RTV of control group and CRTV of treatment group.

### Histology examination

To confirm the combinatorial PTT and chemotherapy efficacy of c(RGDyK)- SNSC-cypate-PTX for tumor therapy, histology analysis of tumor tissues was performed after treatment. Tumor tissues were separated and fixed with 10% neutral buffered formalin and embedded in paraffin (*n* = 8). The sliced organs were stained with H&E and examined under a microscope.

### Statistical analysis

Data was expressed as mean ± standard deviation. Statistical analysis was performed by using students' *t*-test with statistical significance assigned for *P* value of <0.05.

## CONCLUSIONS

In this manuscript, we developed NIR light-triggered micelles that could simultaneously deliver anticancer drug PTX and NIR dye cypate to tumor sites for combined chemo-photothermal therapy. Cypate as a NIR imaging agent not only enhanced the dissociation of the micelles under irradiation, but also revealed high temperature response for PTT. Conjugation of cyclic peptide RGD to the micelle surface enhanced the uptake of the micelles to integrin receptor-positive breast cancer cell line MDA-MB-231, and the results further confirmed that active targeting ability was mediated by integrin receptor. To investigate the anti-tumor effect, we first investigate the *in vitro* drug release of the micelles. Experimental results show that, under exposure of NIR light, the release rate of PTX increased significantly from micelle and exerted significant controlled release effect. *In vitro* and *in vivo* anti-tumor studies revealed that the micelles exhibit an excellent PTT and synergistic chemotherapy of cancer via NIR light-triggered release of PTX from micelles, eventually resulting in decreased cancer recurrence rates. Our results support that this NIR light-triggered targeted therapeutic system has the advantages of precisely locating on tumor sites, sensitive imaging for diagnosis, controlled drug release and accurate therapy. This novel system has huge potential in the new era of combination therapies for tumor treatment.

## SUPPLEMENTARY MATERIALS


