# Supplementary material for: Multifunctional near-infrared light-triggered biodegradable micelles for chemo- and photo-thermal combination therapy
Source: Oncotarget. 2016 Jun 29;7(50):82170–84. doi: 10.18632/oncotarget.10320 (PMC5347683; doi:10.18632/oncotarget.10320)
Supplement: Supplementary file 1 [file oncotarget-07-82170-s001.pdf]

Multifunctional near-infrared light-triggered biodegradable micelles for chemo- and photo-thermal combination therapy

Supplementary Materials

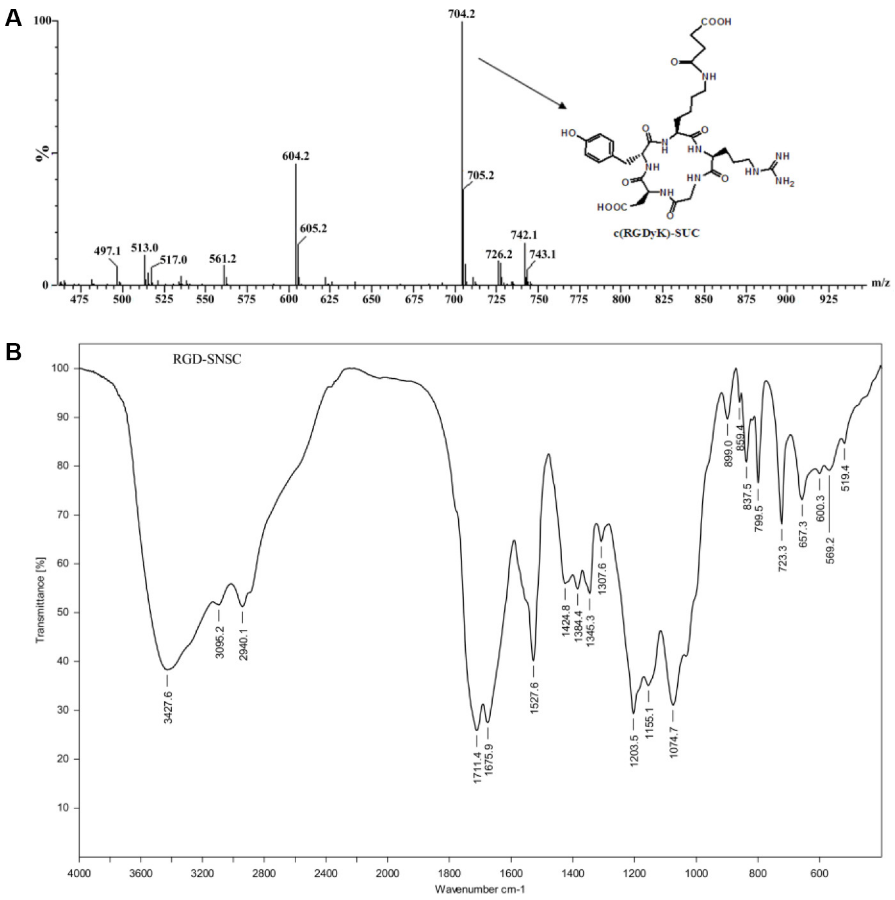

Supplementary Figure S1: (**A**) The mass spectrum of c(RGDyK)-SUC; (**B**) The FTIR spectrum of c(RGDyK)-SNSC.
